# Bcl-2-Dependent Persistence of Mononuclear Phagocytes Promotes Ocular Fibrosis

**DOI:** 10.3390/ijms27167455

**Published:** 2026-08-20

**Authors:** Yong-Seok Song, Shoujian Wang, Soesiawati R. Darjatmoko, Nader Sheibani, Christine M. Sorenson

**Affiliations:** 1 Department of Ophthalmology and Visual Sciences, University of Wisconsin School of Medicine and Public Health, Madison, WI 53705, USA; song224@wisc.edu (Y.-S.S.); shoujianwang@wisc.edu (S.W.); srdarjat@wisc.edu (S.R.D.); 2 McPherson Eye Research Institute, University of Wisconsin School of Medicine and Public Health, Madison, WI 53705, USA; 3 Department of Cell and Regenerative Biology, University of Wisconsin School of Medicine and Public Health, Madison, WI 53705, USA; 4 Department of Pediatrics, University of Wisconsin School of Medicine and Public Health, Madison, WI 53705, USA

**Keywords:** Bcl-2, fibrosis, choroidal neovascularization, proliferative vitreoretinopathy, macrophages, neutrophils, inflammation

## Abstract

Ocular diseases, such as neovascular age-related macular degeneration (nAMD) and proliferative vitreoretinopathy (PVR), have a fibrotic component that negatively impacts vision. Unfortunately, few treatments are available to mitigate fibrosis in the eye. The clearance of inflammatory cells proceeds, at least in part, through the intrinsic cell death pathway in which Bcl-2 family members play integral roles. Here, we assessed the influence of Bcl-2 expression in mononuclear phagocytes (MP) on the engagement and clearance of inflammatory cells, choroidal neovascularization (CNV), and subsequent subretinal fibrosis in a mouse laser-induced CNV model. Lack of Bcl-2 expression in MP (Bcl-2^MP^ mice) decreased neutrophil (Gr1^+^) and microglia (Iba1^+^) presence without impacting M1 (CD80^+^) and M2 (CD206^+^) macrophage presence, CNV, or fibrosis during the first 2 weeks following laser photocoagulation. Later, after inflammation dampens, decreased later-stage fibrosis and CNV were noted in Bcl-2^MP^ mice, which were accompanied by increased presence of M2 macrophages (CD206^+^). However, how these increased levels of CD206^+^ M2 macrophages in the absence of Bcl-2 contribute to decreased CNV and fibrosis remains unknown. To address whether Bcl-2 expression affects other forms of ocular fibrosis, we utilized the dispase PVR model. Bcl-2^MP^ mice, or treatment of wild-type mice with Bcl-2 inhibitors, significantly decreased fibrosis in the PVR model. Furthermore, Bcl-2 inhibitors mitigated CNV and fibrosis (collagen I-defined) in wild-type mice during laser photocoagulation. Thus, inhibition of Bcl-2 activity prevents the late-stage clearance of CD206^+^ M2 macrophages during nAMD and PVR, mitigating ocular neovascularization and fibrosis.

## 1. Introduction

The visual deficit that initially arises from retinal degeneration in dry age-related macular degeneration (AMD) is often complicated by the secondary effects of choroidal neovascularization (CNV) (wet or neovascular AMD; nAMD) [1]. Scarring and atrophy negatively influence vision outcomes in nAMD patients, even those receiving the standard of care treatment, i.e., anti-vascular endothelial growth factor (VEGF) therapies [2]. In the Comparisons of Age-Related Macular Degeneration Treatment Trials (CATT), about 1/3 of patients developed subretinal scars during the first year of anti-VEGF treatment, with an additional 10% of patients forming scars during the second year of treatment [3]. Typically, macular atrophy will develop adjacent to or within the subretinal scar [3]. Thus, gaining a better understanding of the processes involved in subretinal scar formation and its resolution will have a direct impact on vision outcomes for nAMD patients.

Rhegmatogenous retinal detachment (RRD) is the most common surgically treated retinal detachment. Retinal scarring, specifically proliferative vitreoretinopathy (PVR), is the main reason for surgical retinal reattachment failure resulting in vision loss. In cases with severe trauma or penetration to the globe, nearly half of the surgical attempts fail [4,5]. Unfortunately, few, if any, pharmacological treatments are available for PVR. Once established, PVR is difficult to treat and remains the leading cause of recurrent retinal detachment and failure of retinal detachment surgery [6,7,8]. Unfortunately, whether shared cellular and molecular mechanisms facilitate scar formation during PVR and subretinal fibrosis remains largely unknown.

The repair processes that heal damaged tissue can go awry, leading to excessive tissue fibrosis and scar formation. Wound repair due to mechanical damage has been extensively studied in the skin, where pathological fibrosis leads to disfiguring scars, limiting movement. Less is known about pathological fibrosis in the eye. However, the unfortunate endpoint, impaired vision, is all too well known. Although fibrosis occurs in many ocular pathologies with an inflammatory component, the processes that drive the fibrotic development in nAMD and PVR are incompletely understood, leaving treatment options limited.

Ineffective cell turnover disturbs finely orchestrated reparative processes, leading to persistent inflammation and pathologic fibrosis. Members of the Bcl-2 family positively or negatively influence cell loss in most cell types, including myeloid cells. Bcl-2 family members are effective immune cell regulators that modulate cell death through the intrinsic, mitochondrial, or Bcl-2-regulated pathways [9,10]. Bcl-2 curtails cell death by keeping multiple-BH-domain family members, including Bim, in an inactive state which is upended with a death stimulus and the appearance of BH3-domain-only proteins [11]. This teeter-totter balance of family members is disrupted when optimal amounts or function of these proteins are not available due to single nucleotide polymorphisms or posttranscriptional and/or posttranslational modifications. Thus, understanding how Bcl-2 family member expression impacts tissue repair processes will contribute to novel uses for known modulators of their expression.

Bcl-2 extends the lifespan of macrophages [12] and is a key contributor to the fibrosis and scarring in idiopathic pulmonary fibrosis (IPF) and hypertrophic scarring [13,14,15]. Bcl-2 expression also supports the recruitment and polarization of M2 macrophages driving melanoma tumor formation in vivo [16]. However, the role Bcl-2 plays in ocular macrophage homeostasis and dysregulation during fibrotic processes would benefit from further exploration. We recently showed that the lack of Bim expression in ocular mononuclear phagocytes (MP) increases subretinal fibrosis, potentially due to sustained MP infiltration following laser photocoagulation [17]. Whether Bcl-2 tempers Bim to modulate MP lifespan and ocular fibrosis has yet to be determined. Here, utilizing murine models, we assessed the impact of Bcl-2 expression in MP on the development of ocular fibrosis, specifically subretinal fibrosis and PVR.

## 2. Results

### 2.1. Decreased Subretinal Fibrosis in Bcl-2^MP^ Mice

**Figure 1:**
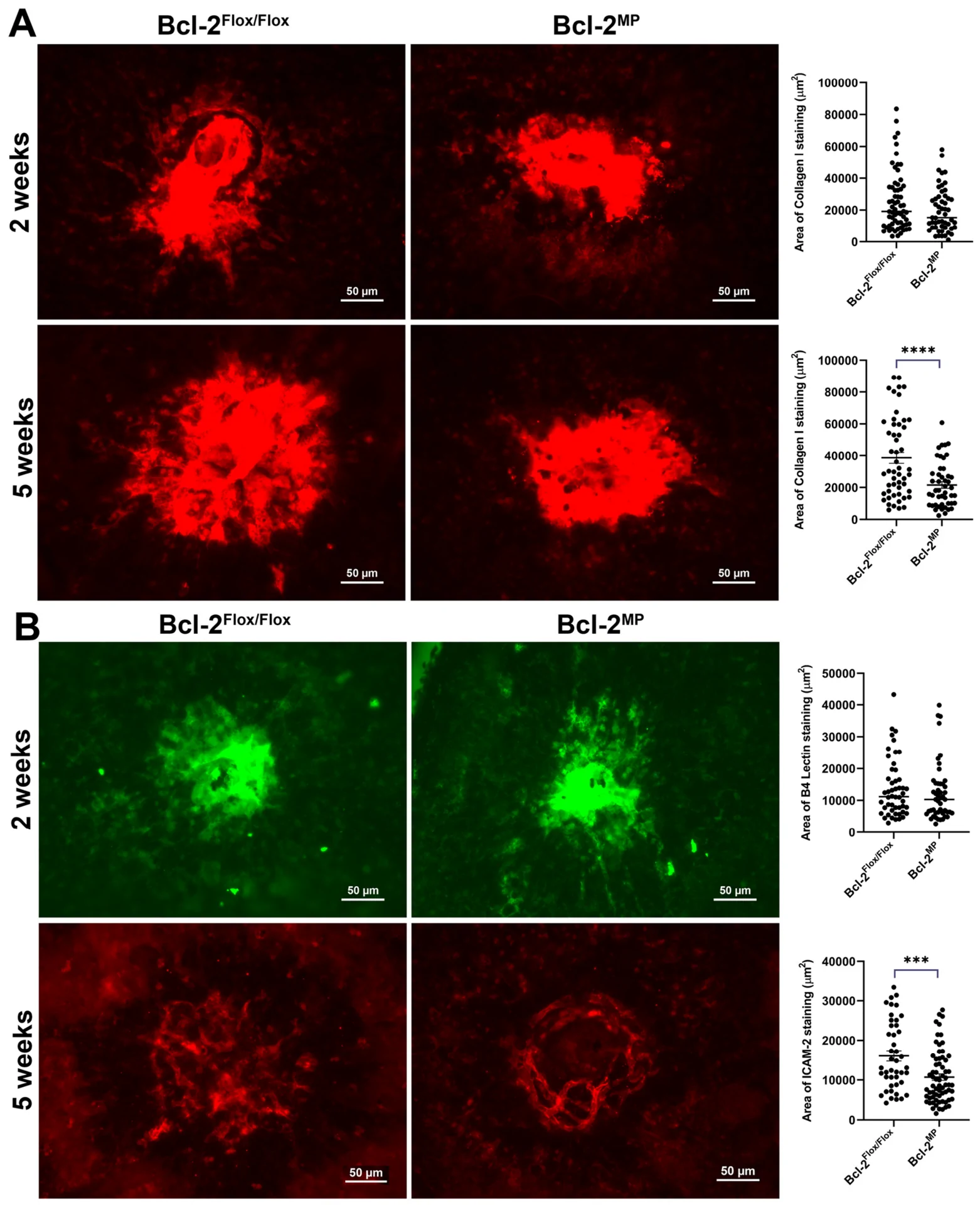
Lack of Bcl-2 in MP decreases subretinal fibrosis and CNV. Bcl-2^Flox/Flox^ and Bcl-2^MP^ mice (3-month-old) underwent laser photocoagulation. The choroid/RPE was isolated 2 and 5 weeks later and wholemount stained with anti-collagen I (fibrosis; Red) (**A**). To assess CNV, choroid/RPE was wholemount stained with Isolectin B4 (2 weeks; Green) or anti-ICAM-2 (5 weeks; Red) (**B**). Representative images of staining are shown with the area of staining quantified on the right; 10 mice were used for each condition. Scale bar = 50 µm. Quantitative data are presented as mean ± SD. The statistical evaluations were performed as detailed in Methods. A *p*-value of less than 0.05 is considered significant (*p* = 0.0825, **** *p* = 0.00004, *p* = 0.4769, *** *p* = 0.0003, respectively).

Myeloid cells, such as mononuclear phagocytes (MP), play an important role in establishing subretinal fibrotic scars [17,18]. Bcl-2 and Bim modulate the lifespan of many cell types, including MP, with Bcl-2 extending lifespan and Bim aiding their turnover [12,19]. To ascertain the contribution of Bcl-2 expression in MP to the development of subretinal fibrosis and/or choroidal neovascularization (CNV), we utilized mice lacking Bcl-2 expression in MP (Bcl-2^MP^ mice). The relative area of the subretinal fibrosis was assessed by staining with anti-collagen I at 2-weeks following laser photocoagulation, when maximum CNV is noted, and later, when inflammation has dwindled (5 weeks following laser; later-stage fibrosis). CNV levels were assessed by Griffonia Simplicifolia Lectin I (GSL I; Isolectin B4) staining at 2 weeks (Green) or anti-ICAM-2 staining (Red) at 5 weeks. Isolectin B4 staining of the vascular lesions is ineffective at 5 weeks post-laser because of changes in epitope recognition on endothelial cells due to excessive tissue remodeling. Two weeks following laser photocoagulation, no significant difference in subretinal fibrosis (defined by collagen I staining; Red) or CNV (defined by Isolectin B4 staining; Green) was noted between Bcl-2^MP^ mice and their control littermates (Bcl-2^Flox/Flox^; Figure 1A,B). However, after 5 weeks following laser photocoagulation, Bcl-2^MP^ mice demonstrated a significant decrease in subretinal fibrosis (defined by collagen I staining; Red) and CNV (defined by ICAM-2 staining; Red) compared to the results for their Bcl-2^Flox/Flox^ littermates (Figure 1A,B; lower panels).

To ensure the accuracy of our findings, we also performed mixed analysis using the data for Figure 1B (5-week time point) as an example. After exclusion of prespecified outliers, 105 lesion-level observations from 26 mice were included in the mixed-effects analysis. Bcl-2^MP^ mice had significantly lower CNV lesion measurements than those of the control Bcl-2^Flox/Flox^ mice on the log scale (β = −0.498, SE = 0.122, *p* = 0.0006), adjusting for sex and accounting for clustering within mice. This corresponds to an estimated Bcl-2^MP^ to Bcl-2^Flox/Flox^ ratio of 0.61 (95% CI, 0.47–0.78), or approximately 39% lower values in the knockout group and an adjusted standardized mean difference of approximately d = −0.87 (95% CI, −1.34 to −0.40). Sex was not significantly associated with the outcome (*p* = 0.08). The sensitivity analysis using the unpaired *t*-test produced the same overall conclusion and was used throughout the manuscript.

### 2.2. Decreased F4/80 Staining in RPE/Choroid from Bcl-2^MP^ Mice

**Figure 2:**
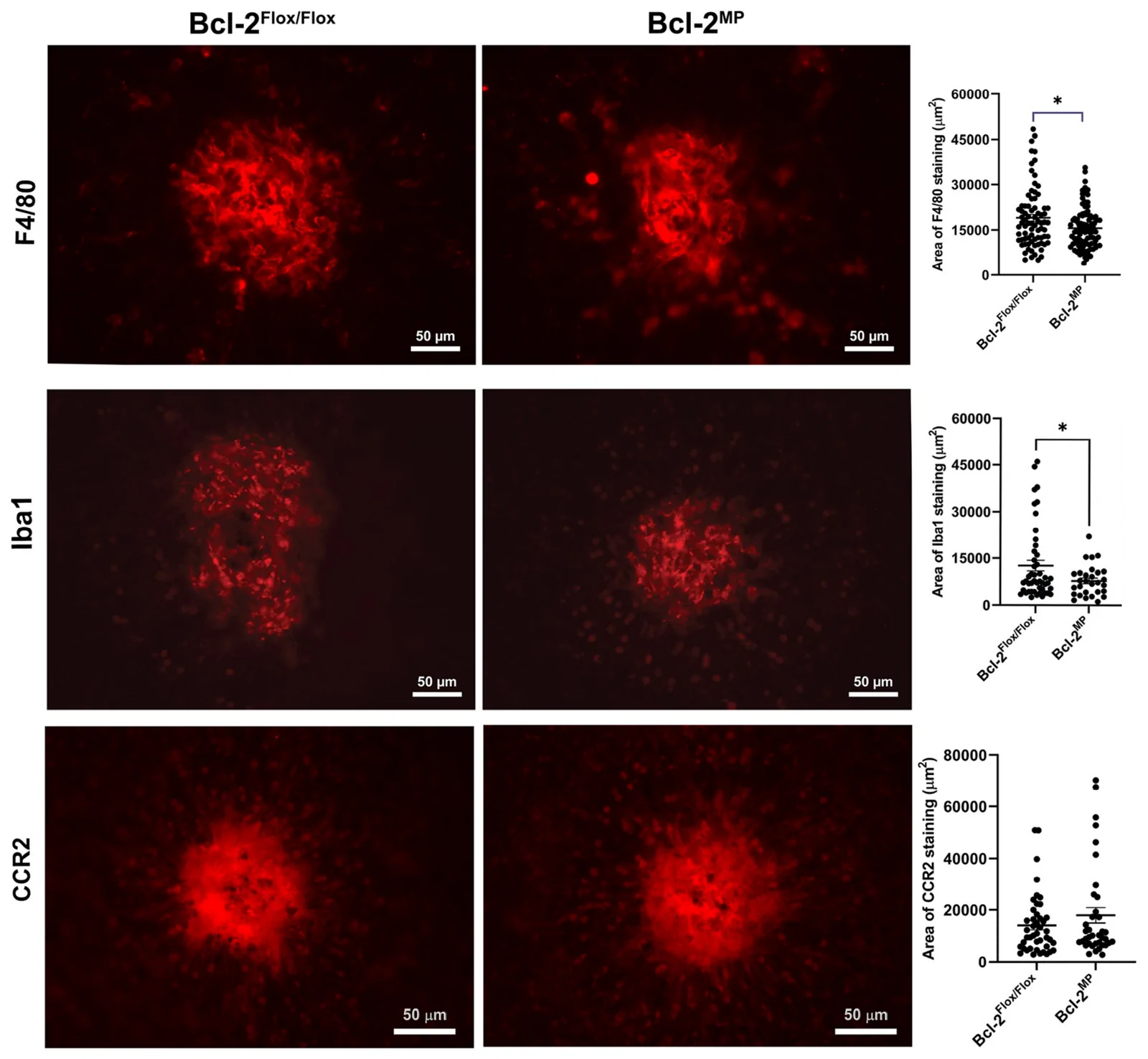
Bcl-2^MP^ mice have decreased F4/80 and Iba1-positive cells in the laser-induced lesions. Bcl-2^Flox/Flox^ and Bcl-2^MP^ mice (3-month-old) underwent laser photocoagulation. Six days later, the choroid/RPE was wholemount stained with anti-F4/80 (pan-MP marker), anti-Iba1, or anti-CCR2, and captured images were quantified using ImageJ. Experiments were performed with 10 mice for each condition. Scale bar = 50 µm. Quantitative data are presented as mean ± SD. The statistical evaluations were performed as detailed in Methods. A *p*-value of less than 0.05 is considered significant (* *p* = 0.0102, * *p* = 0.0408, *p* = 0.2609, respectively).

Bcl-2 is a critical antagonist of Bim in lymphocytes. The consequences of not maintaining an appropriate Bcl-2 to Bim ratio in lymphocytes are well characterized regarding cellular homeostasis [20,21,22]. Cell loss facilitates MP removal from various locations [23]. However, less is understood regarding the effect Bcl-2 has on MP in the choroid. Here, we addressed whether lack of Bcl-2 influenced MP resolution following laser photocoagulation by assessing F4/80 staining (pan microglia and MP marker). A significant decrease in F4/80 staining was noted in the choroid/RPE lesions from Bcl-2^MP^ mice 6 days following laser photocoagulation compared to the results for their Bcl-2^Flox/Flox^ littermates (Figure 2; top panels).

Next, Bcl-2^MP^ mice underwent laser photocoagulation, and microglia levels in the lesions were specifically assessed by Iba1 staining. Figure 2 (middle panels) shows decreased Iba1 staining in the choroid/RPE lesions from Bcl-2^MP^ mice when compared to their Bcl-2^Flox/Flox^ littermates. Given that the C–C chemokine receptor type 2 (CCR2) marks systemic monocytes recruited to the site of injury, we also assessed its expression in these lesions following laser photocoagulation. Although F4/80 and Iba1 staining levels decreased in Bcl-2^MP^ mice, similar CCR2 staining levels were noted in both Bcl-2^Flox/Flox^ and Bcl-2^MP^ mice following laser photocoagulation (Figure 2; lower panels). Thus, systemic recruitment of CCR2-positive cells to the lesion sites was not influenced by Bcl-2 expression in MP.

### 2.3. Lack of Bcl-2 Expression Increased the Abundance of CD206^+^-Myeloid Cells in CNV Lesions

**Figure 3:**
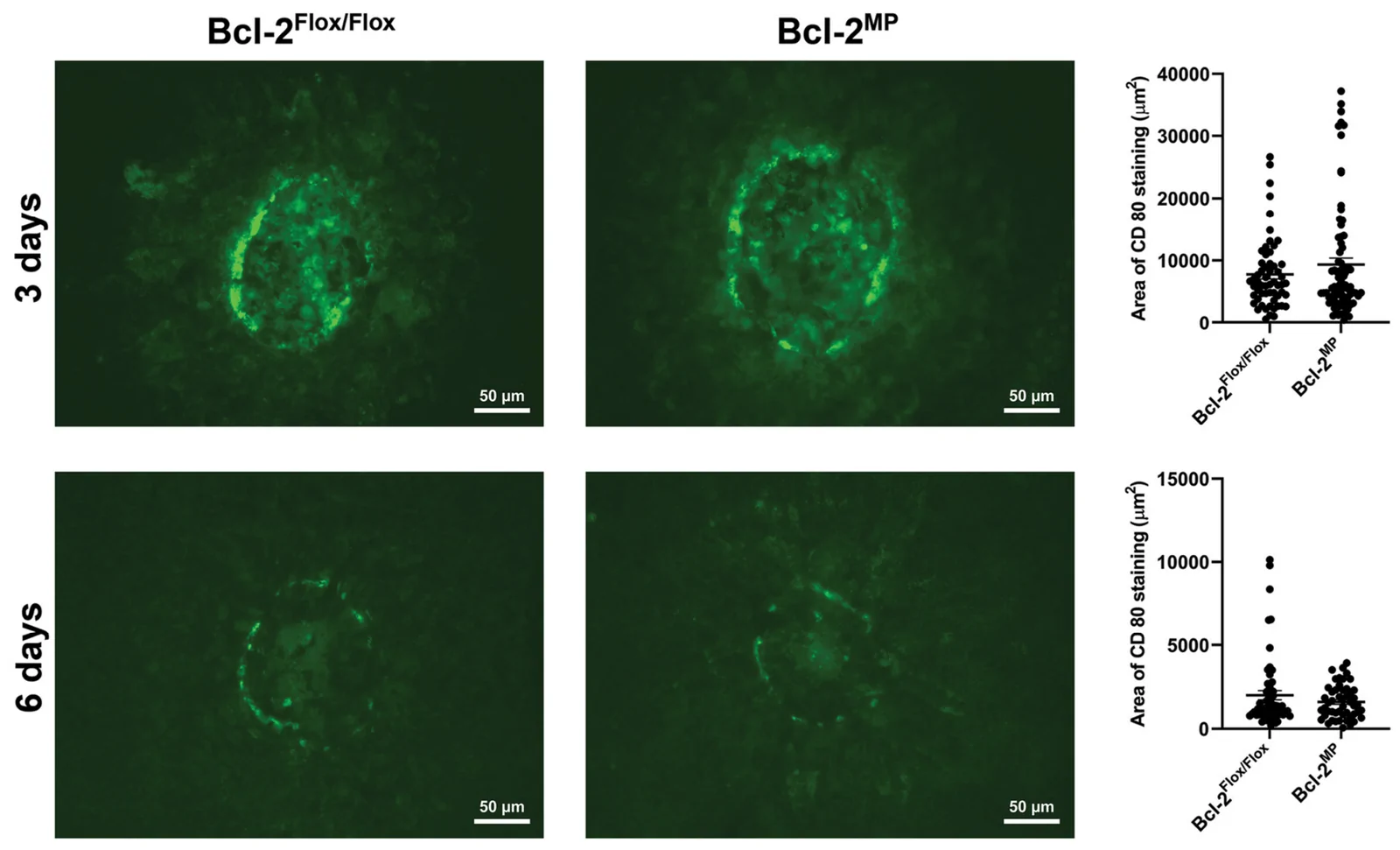
Similar resolution of CD80^+^-myeloid cells in Bcl-2^Flox/Flox^ and Bcl-2^MP^ mouse CNV lesions. The choroid/RPE from Bcl-2^Flox/Flox^ and Bcl-2^MP^ mice (3-month-old) subjected to laser photocoagulation was harvested and wholemount stained with anti-CD80. Captured images were quantified after 3 and 6 days using ImageJ. These experiments were performed with 10 mice for each condition. Scale bar = 50 µm. Quantitative data are presented as mean ± SD. The statistical evaluations were performed as detailed in Methods. A *p*-value of less than 0.05 is considered significant (*p* = 0.256, *p* = 0.2298, respectively).

Macrophages can be grossly grouped into M1 (pro-inflammatory) and M2 (anti-inflammatory) subgroups. M1 macrophages, when activated, produce proinflammatory cytokines, while M2 macrophages produce anti-inflammatory cytokines and impact fibrosis [24,25]. Given the enhanced resolution of F4/80-positive cells in mice lacking Bcl-2 expression in MP, we assessed markers CD80 (M1) and CD206 (M2) following laser photocoagulation. M1 macrophages aid in the clearance of foreign organisms or substances and are recruited and swiftly cleared. Figure 3 shows similar levels of CD80^+^ myeloid cells (M1 marker) at 3 and 6 days following laser photocoagulation in Bcl-2^Flox/Flox^ and Bcl-2^MP^ mouse lesions.

**Figure 4:**
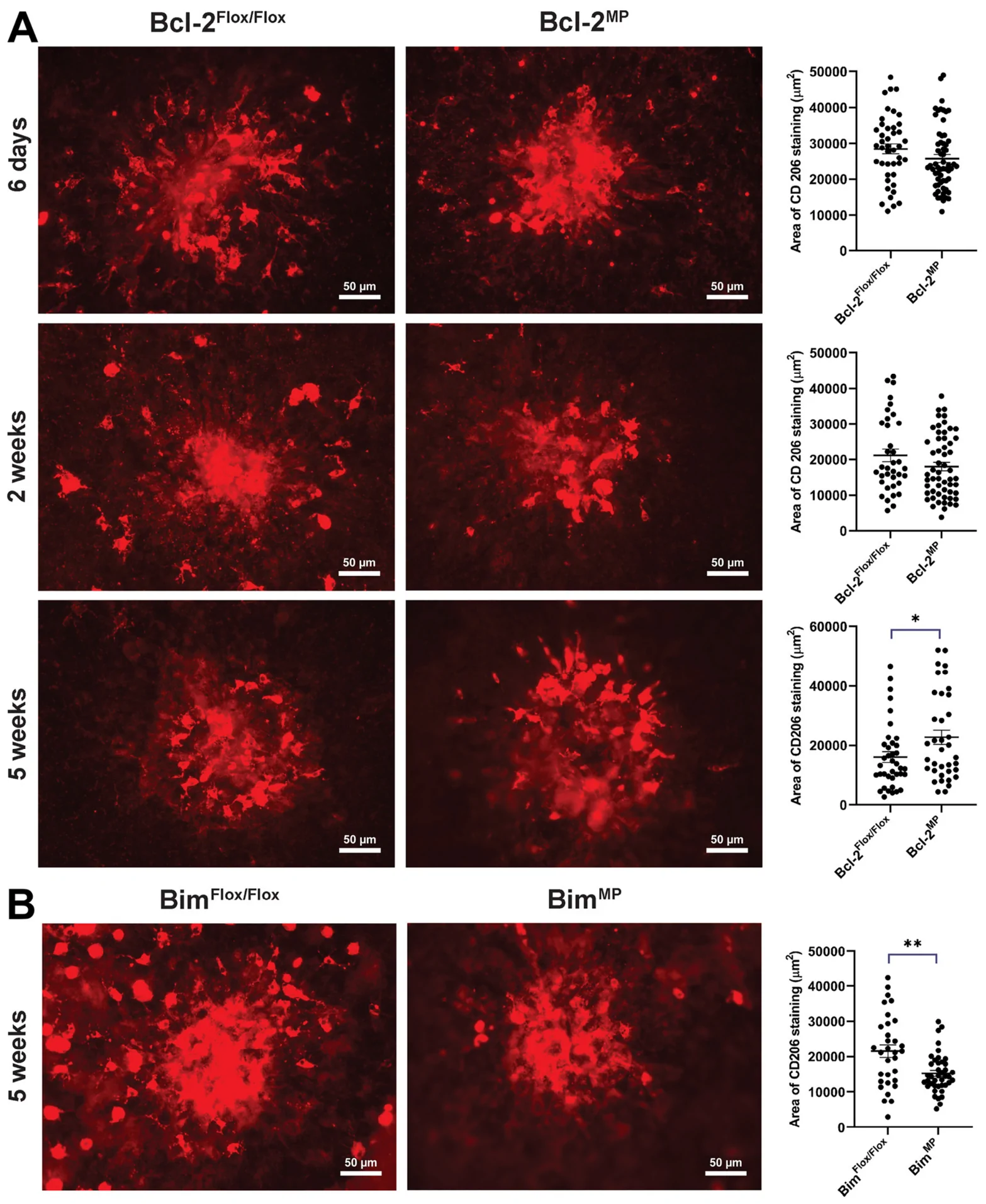
Bcl-2^MP^ mice display increased CD206^+^ myeloid cells 5 weeks post-laser. In Panel (**A**), Bcl-2^Flox/Flox^ and Bcl-2^MP^ (3-month-old) mice underwent laser photocoagulation, with the choroid/RPE harvested and stained with anti-CD206 6 days, 2 weeks, and 5 weeks post-laser. The lesion area in captured images was quantified using ImageJ. In Panel (**B**), Bim^Flox/Flox^ and Bim^MP^ mice underwent laser photocoagulation, and 5 weeks later, the choroid/RPE was harvested and stained with anti-CD206. The lesion area in captured images was quantified using ImageJ. These experiments were performed with 10 mice for each genotype. Scale bar = 50 µm. Quantitative data are presented as mean ± SD. The statistical evaluations were performed as detailed in Methods. A *p*-value of less than 0.05 is considered significant (*p* = 0.1364, *p* = 0.1322, * *p* = 0.0279, ** *p* = 0.0011, respectively).

Next, we examined CD206 levels (M2 marker) in CNV lesions. Unlike CD80^+^ myeloid cells, which were cleared considerably by 6 days, substantial levels of CD206^+^ myeloid cells persisted in these lesions through 5 weeks following laser photocoagulation (Figure 4A). Bcl-2^Flox/Flox^ and Bcl-2^MP^ mice demonstrated similar levels of CD206 staining at 6 days and 2 weeks following laser photocoagulation. However, 5 weeks after laser photocoagulation, increased levels of CD206^+^ myeloid cells were noted in choroid/RPE lesions from Bcl-2^MP^ mice compared to the levels for Bcl-2^Flox/Flox^ mice (Figure 4A, lower panels). Since Bim acts in an antagonistic manner towards Bcl-2, we next assessed CD206 staining in Bim^Flox/Flox^ and Bim^MP^ mice, which were not previously examined. These results showed decreased levels of CD206^+^ myeloid cell staining in Bim^MP^ mice 5 weeks following laser photocoagulation (Figure 4B). Thus, Bcl-2 and Bim expression in MP impacts the later-stage resolution of CD206-positive myeloid cells from laser-induced lesions and keeps inflammatory processes in check. In future experiments, further confirmation of the identity of M1 and M2 macrophages using other markers will provide additional support for the exact identity of these macrophages impacted by the dysregulation of Bcl-2/Bim levels.

### 2.4. Decreased Aboundance of Gr1^+^-Neutrophils in the CNV Lesions of Bcl-2^MP^ Mice

**Figure 5:**
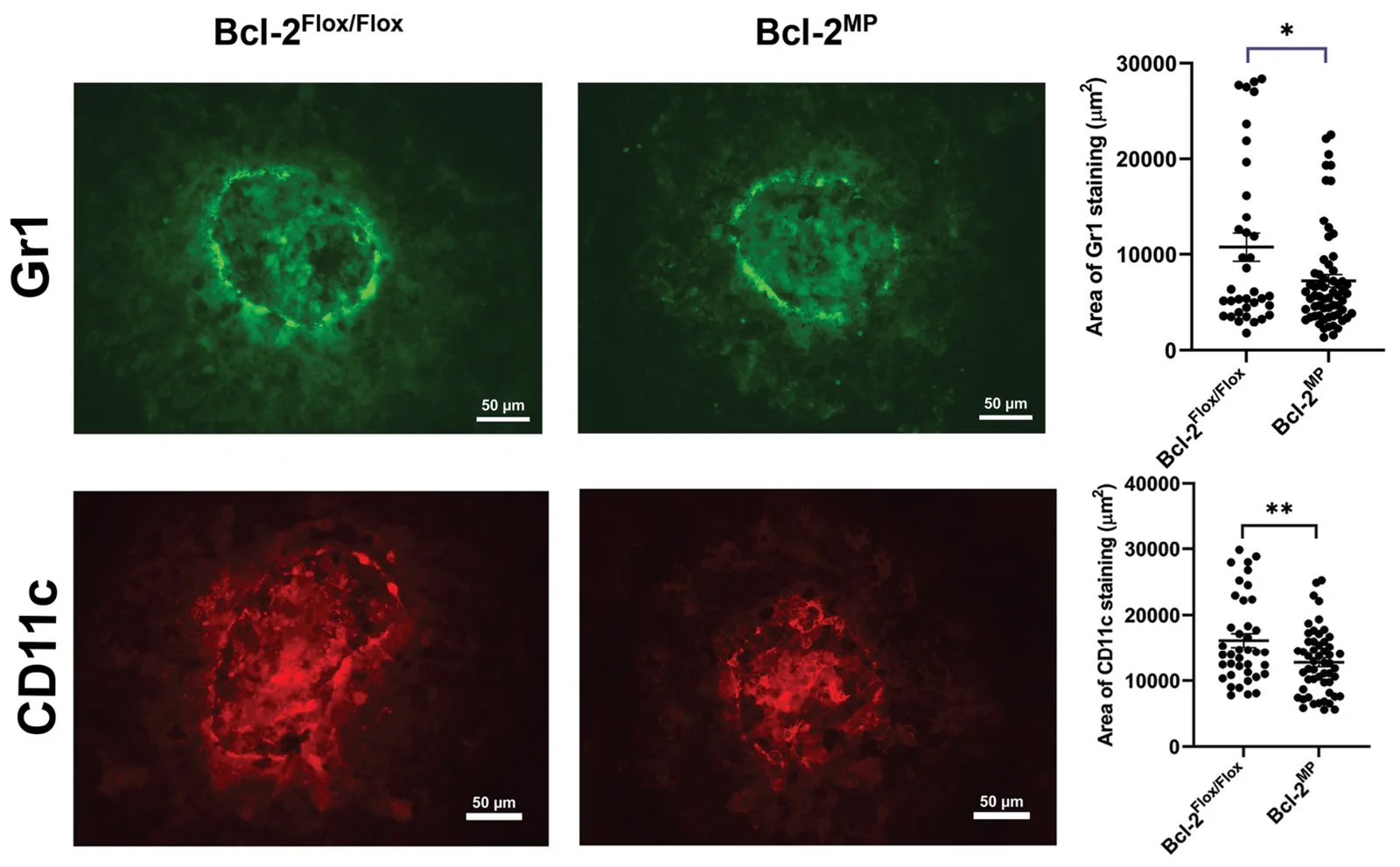
Decreased Gr1^+^ (Green) and CD11c^+^ (Red) following laser photocoagulation in Bcl-2^MP^ mice. Bcl-2^Flox/Flox^ and Bcl-2^MP^ mice (3-month-old) were subjected to laser photocoagulation. Three days later, the choroid/RPE was harvested and stained with anti-Gr1 (Green) or anti-CD11c (Red). The area of lesion staining was quantified in captured images using ImageJ. These experiments were carried out using 10 mice for each condition. Scale bar = 50 µm. Quantitative data are presented as mean ± SD. The statistical evaluations were performed as detailed in Methods. A *p*-value of less than 0.05 is considered significant (* *p* = 0.0148, ** *p* = 0.0063, respectively).

Neutrophils (Ly6c^high^Ly6g^+^; [26]) are recruited early to a site of injury, forming a ring around the wound edge, preventing infection from contaminating microbes. During this fight, the tissue can also be injured, with neutrophils contributing to inflammation, as well as to subsequent tissue remodeling and fibrosis. Decreasing neutrophil levels have been shown to reduce fibrosis [27]. The levels of neutrophil recruitment to the lesions were significantly reduced 3 days following laser photocoagulation, as assessed by mouse anti-Gr1 (anti-mouse Ly6g/Ly6c clone RB6-8C5 identifies peripheral blood neutrophils and depletes granulocytes in vivo) staining in Bcl-2^MP^ mice (Figure 5, top panels). Although anti-Ly6g specifically targets the neutrophil, it suffers from lower efficiency than that of anti-Gr1 in depleting neutrophils [28]. Thus, confirmation of our anti-Gr1 findings using the more neutrophil-specific marker (anti-Ly6g) may yield novel findings in future studies.

CD11c/*Itgax* is a member of the β2 integrin family and is most strongly associated with dendritic cells. It is also expressed on several other immune cell types, including neutrophils. Although CD11c^+^ macrophages are essential for CNV, the contribution of dendritic cells to CNV is very limited [29,30]. Neutrophils express moderate levels of CD11c, which increase with age/activation [31]. In addition, lack of CD11c impairs neutrophil maturation and decreases CNV [29,32]. Bcl-2^MP^ mice also had significantly lower levels of CD11c^+^ myeloid cells in their lesions compared with those of their Bcl-2^Flox/Flox^ littermates following laser photocoagulation (Figure 5, lower panels). Thus, Bcl-2 expression in MP influences neutrophil, and likely, proangiogenic macrophage, recruitment to CNV lesions.

### 2.5. Bcl-2 Inhibition Decreased CNV and Fibrosis in Wild-Type Mice

Since Bcl-2 extends a cell’s lifespan, much focus has been given to identifying inhibitors. Bcl-2 inhibitors, such as ABT199 (Venetoclax), are efficacious in treating various malignancies [33]. These inhibitors are typically BH3 domain mimetics that disrupt Bcl-2′s interaction with death effector family members, unleashing their proapoptotic activity [22]. Normal ocular tissues generally do not rely solely on Bcl-2 and express redundant pro-survival proteins, including Bcl-xl and Mcl-1, reducing their dependence on Bcl-2. In contrast, hematopoietic cells are highly dependent on Bcl-2, as observed in hematological malignancies and their selectiveness for Bcl-2 inhibition [34]. ABT199 is highly selective for Bcl-2, with minimal activity against Bcl-xl, reducing the possibility of off-target toxicities in tissues where other anti-apoptotic proteins predominate.

**Figure 6:**
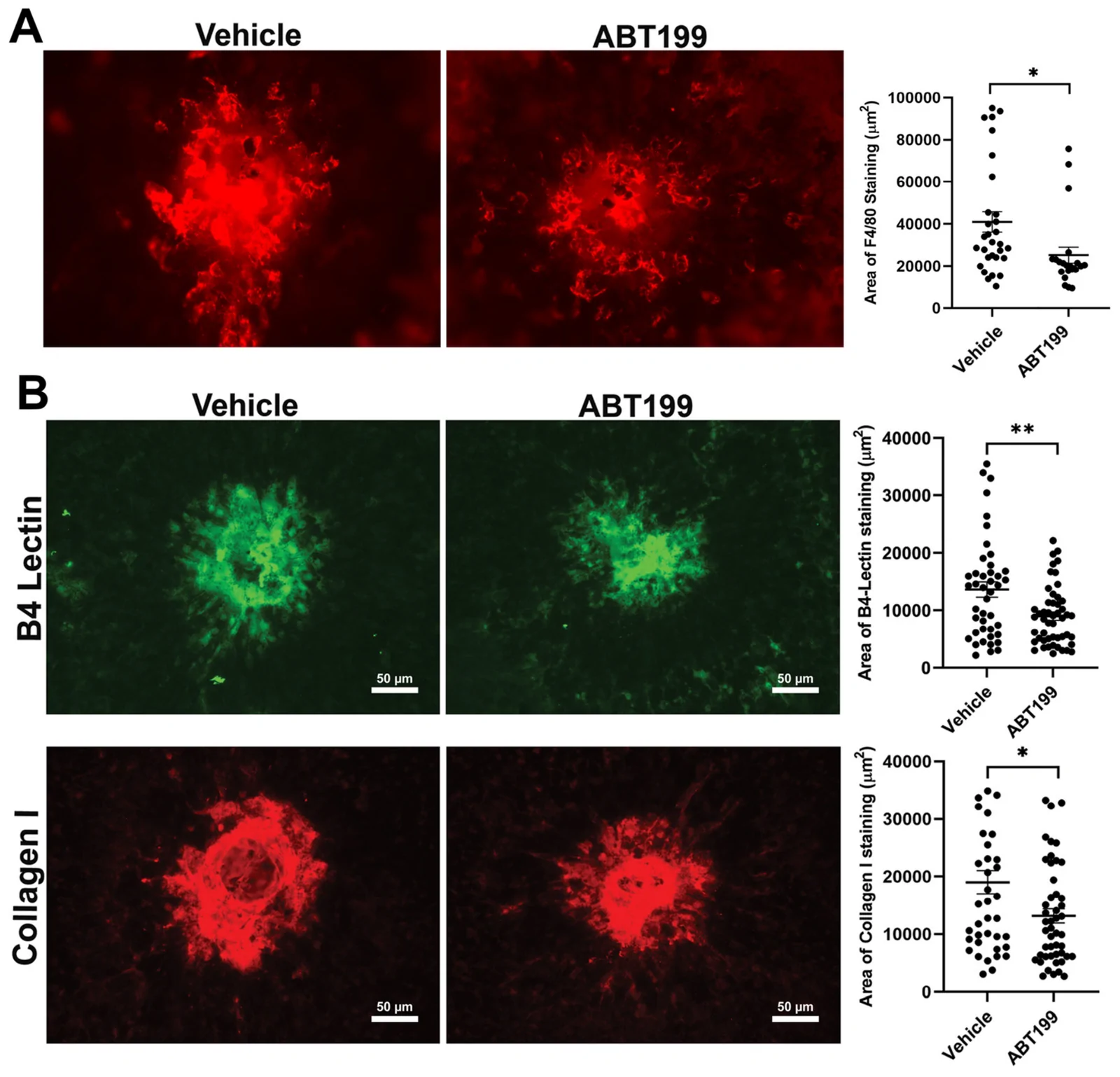
ABT199 decreased CNV and fibrosis in wild-type mice. In Panel (**A**), C57BL/6J mice (3-month-old) received intravitreal injection (1 µL) of either vehicle (DMSO) or ABT199 (100 µM; prepared in DMSO) per eye on the day of laser photocoagulation. The choroid/RPE was harvested 6 days following laser photocoagulation and stained with anti-F4/80 (Red). In Panel (**B**), C57BL/6J mice (3-month-old) received intravitreal injection (1 µL) of either vehicle (DMSO) or ABT199 (100 µM prepared in DMSO) per eye on the day of laser photocoagulation and 7 days later. Choroid/RPE harvested 14 days after laser photocoagulation was immunostained with Isolectin B4 (Green) and anti-collagen I (Red). The area of lesion staining was quantified in captured images using ImageJ. These experiments were performed with 10 mice for each condition. Scale bar = 50 µm. Quantitative data are presented as mean ± SD. The statistical evaluations were performed as detailed in Methods. A *p*-value of less than 0.05 is considered significant (* *p* = 0.0125, ** *p* = 0.0022, * *p* = 0.0124, respectively).

**Figure 7:**
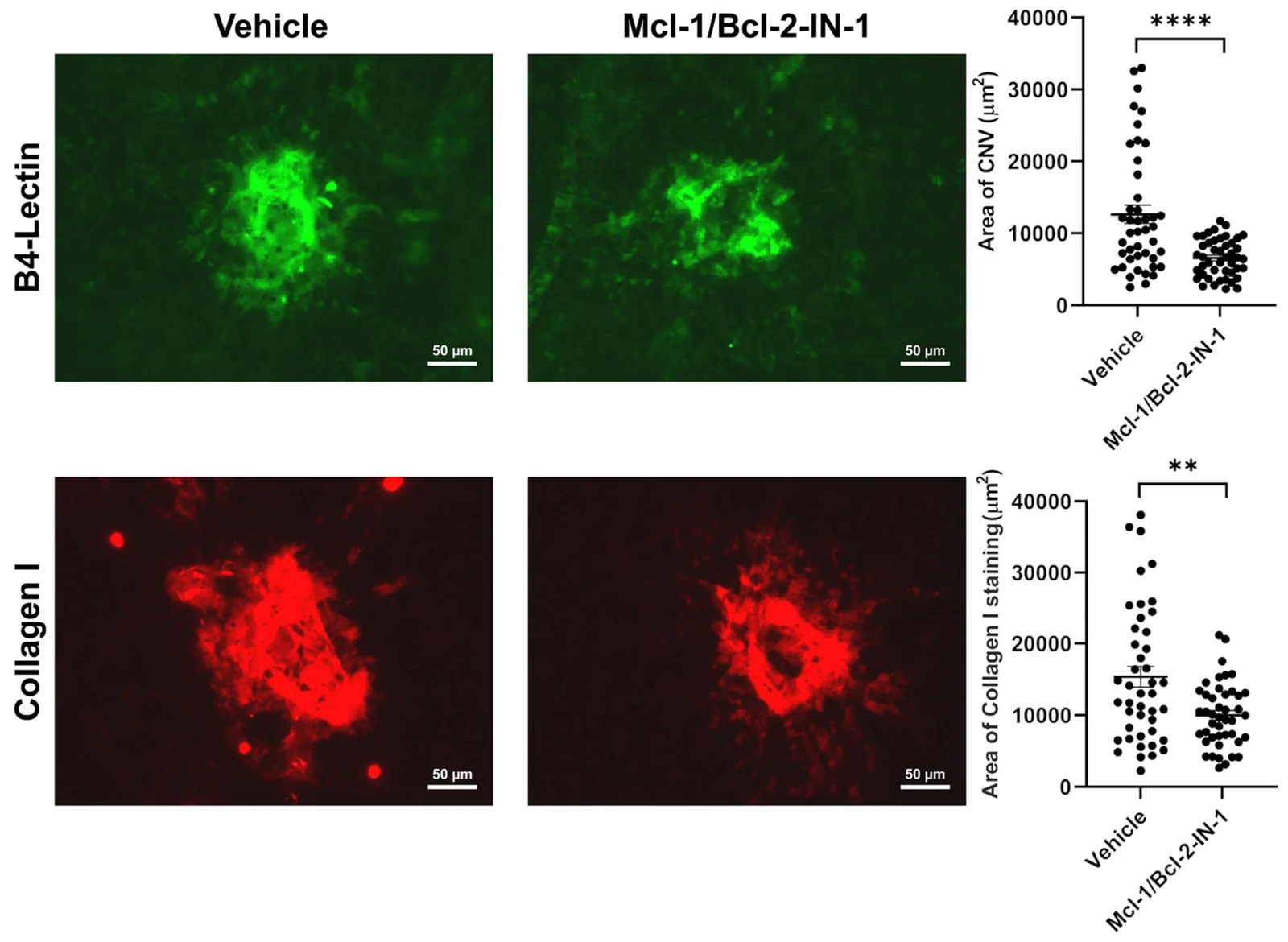
Mcl-1/Bcl-2-IN-1 decreased CNV and fibrosis in wild-type mice. C57BL/6J mice (3-month-old) received an intraperitoneal (IP) injection of vehicle (10% DMSO, 30% PEG 400, 7.5% Tween 20, 7.5% Tween 80, and 45% sterile H_2_O) or Mcl-1/Bcl-2-IN-1 (15 mg/kg, prepared using the vehicle mixture) starting 2 days prior to laser photocoagulation and then 2, 6 and 10 days following laser photocoagulation. Fourteen days following laser photocoagulation, the harvested choroid/RPE was stained with Isolectin B4 (Green) and anti-collagen I (Red). The lesion area in captured images was quantified using ImageJ. These experiments were performed with 10 mice for each condition. Scale bar = 50 µm. Quantitative data are presented as mean ± SD. The statistical evaluations were performed as detailed in Methods. A *p*-value of less than 0.05 is considered significant (**** *p* = 0.00002, ** *p* = 0.0011, respectively).

Here, we examined the effectiveness of Bcl-2 inhibitor ABT199 on CNV and fibrosis. ABT199 significantly reduced the levels of F4/80^+^ cells in C57BL/6J mouse CNV lesions following laser photocoagulation (Figure 6A) [35,36]. To further determine the specific role of Bcl-2 family member anti-apoptotic proteins in modulating CNV and fibrosis, we also evaluated the activity of Mcl-1/Bcl-2-IN-1 (also called Nap-1, which inhibits both Mcl-1 and Bcl-2) in our preclinical model. Unlike ABT199, Mcl-1/Bcl-2-IN-1 is not a clinical drug, and little is known regarding its animal or clinical toxicity. Although ocular toxicity has not emerged as a consistent target-organ toxicity by selective inhibitors of Bcl-2 and Mcl-1, such as ABT199 and AZD5991 [37], the impact of Mcl-1/Bcl-2-IN-1 ocular toxicity remains unknown. ABT199, as well as the dual Mcl-1/Bcl-2-IN-1 inhibitor, effectively reduced the levels of both subretinal fibrosis and CNV following laser photocoagulation (Figure 6B and Figure 7). However, these inhibitors may directly affect other ocular non-myeloid cells, which requires verification.

### 2.6. Decreased PVR in Bcl-2^MP^ Mice and with Bcl-2 Inhibitors

**Figure 8:**
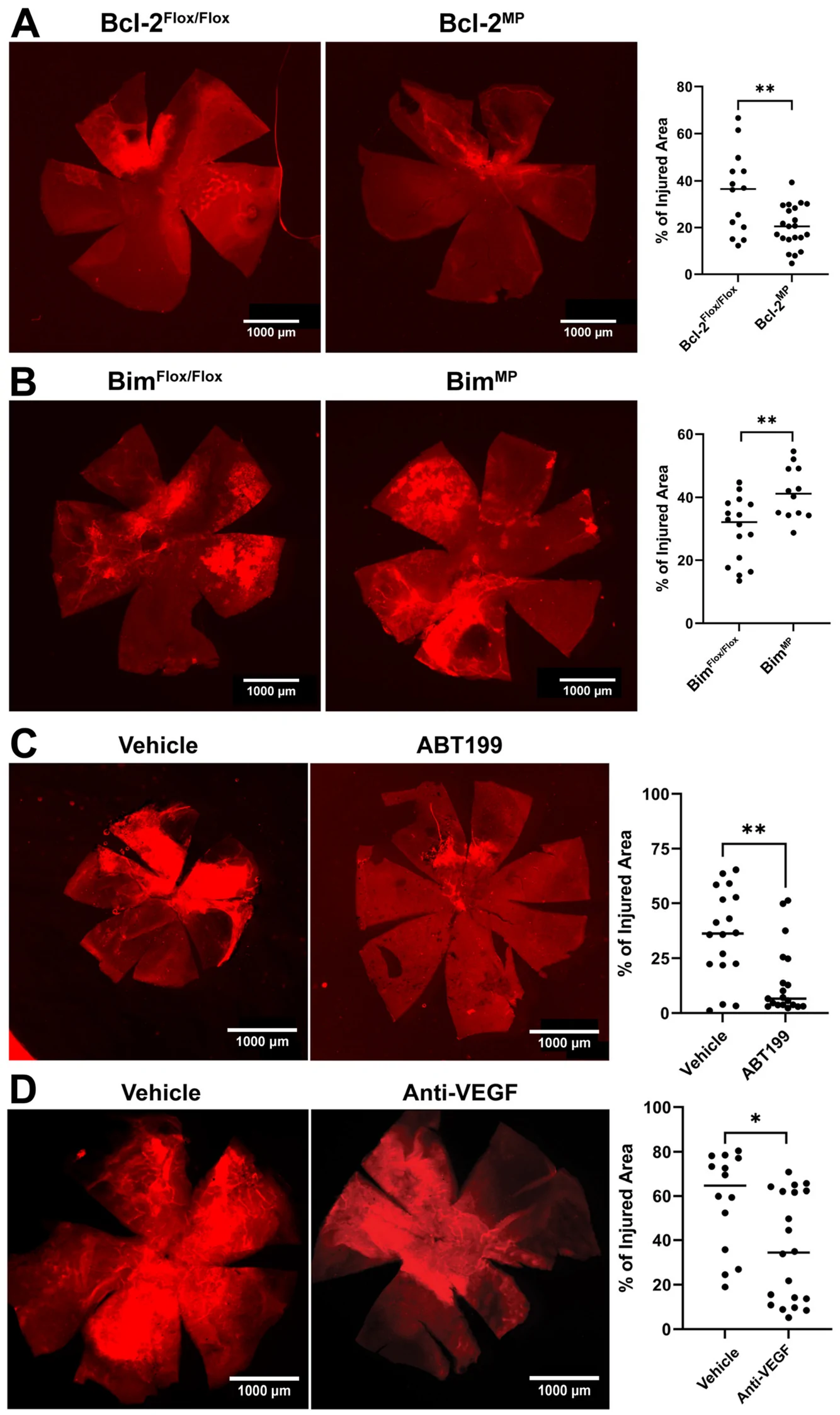
Bcl-2 and Bim expression influence PVR. Conditional Bcl-2 (**A**) and Bim mice (**B**) (3-month-old) received an intravitreal injection of dispase. Retinas were harvested 1 week later. Scale bar = 1000 µm. For Bcl-2 mimetic studies (**C**), C57BL/6J mice (3-month-old) received an intravitreal injection of dispase (0.0015624 units in 1 μL), with selected treatment of either vehicle (DMSO; 1 μL) or ABT199 (100 µM prepared in DMSO; 1 μL) per eye on Day 0. Mice received a second dose of either vehicle (DMSO) or ABT199 5 days later. Two weeks following dispase administration, the retinas were harvested. For anti-VEGF treatment (**D**), mice received an intravitreal injection of anti-VEGF (25 ng in 1 μL prepared in saline) or vehicle 2 days prior to dispase injection. Eyes were harvested 1 week after dispase injection and stained with anti-collagen I antibody. The lesion area of captured images was quantified using ImageJ. These experiments were performed with 10 mice for each condition. Scale bar = 1000 µm. Quantitative data are presented as mean ± SD. The statistical evaluations were performed as detailed in Methods. A *p*-value of less than 0.05 is considered significant (** *p* = 0.0017, ** *p* = 0.0032, ** *p* = 0.0011, * *p* = 0.0132, respectively).

To assess whether the influence of Bcl-2 and Bim on fibrosis was unique to the laser photocoagulation model (this study and [17]), we next utilized a murine dispase model of PVR to assess their impact during retinal scar formation. Retinal damage was induced in Bcl-2^MP^ and Bim^MP^ mice with an intravitreal dispase injection, and the area of retinal fibrosis was assessed by collagen I staining, as described above. Bcl-2^MP^ mice demonstrated decreased levels of retinal fibrosis (collagen I staining), while Bim^MP^ mice showed increased levels of retinal fibrosis compared to those of their respective floxed counterparts (Figure 8A,B). Thus, MP expression of Bcl-2 or Bim also impacts retinal scarring, as determined by collagen I staining.

To assess whether Bcl-2 inhibitors effectively mitigate fibrosis in wild-type mice, we next examined the utility of ABT199 for the treatment of PVR in C57BL/6J mice. Previous studies showed the efficacy of Bcl-2 inhibitors in idiopathic pulmonary fibrosis (IPF) treatment for new or established fibrosis [13,38]. To maximize the ability of ABT199 to prevent fibrosis, wild-type mice received two doses of ABT199, one at the time of dispase administration and another 5 days later. Decreased levels of PVR (defined by collagen I staining) were noted in mice receiving ABT199 treatment compared to those of the vehicle control (Figure 8C). Although we did not evaluate the regression of established CNV and PVR fibrosis in our models by ABT199, future experiments are planned to assess the efficacy of ABT199 in producing the regression of fibrosis associated with CNV and PVR.

Since Bcl-2 and VEGF influence each other’s expression, we next assessed the ability of anti-VEGF to impact retinal fibrosis in the PVR model. Figure 8D shows that intravitreal injection of anti-VEGF also significantly decreased the PVR levels (defined by collagen I staining) compared to those of the vehicle control. Thus, modulating Bcl-2 expression and/or activity impacts PVR.

## 3. Discussion

Fibrosis due to injury or disease has a well-documented negative impact on vision. Unfortunately, therapeutic treatments for ocular fibrosis are extremely limited, in part due to our lack of understanding of the processes involved. One of the earliest responders to stress and tissue damage are the neutrophils, which form a ring around the affected tissue. This can be beneficial or detrimental to the healing and repair processes, depending on the circumstances. Neutrophils aid damaged tissue removal and repair by releasing proteolytic enzymes, promoting the formation of new vasculature and repair by the release of growth factors and collagen deposition [39,40,41]. However, this intricately orchestrated repair process goes awry with increased neutrophil activity and/or numbers, leading to enhanced remodeling and scar formation.

In the laser photocoagulation CNV model, Gr1^+^ cells were typically confined to the wound edge in wild-type mice. Bcl-2^MP^ mice displayed lower levels of Gr1^+^ cells along the wound edge. In contrast, lack of Bim expression (globally or in MP) results in dispersion of Gr1^+^ cells throughout the entire site of injury (Supplementary Figure S1), correlating with the increased levels of fibrosis we previously noted in these mice [17]. Lack of CD11c prevents neutrophil maturation and reduces CNV [29,32]. Here, we showed decreased levels of CD11c^+^ cells in the absence of Bcl-2 MP expression. Thus, lack of Bcl-2 expression in MP impacts not only the levels of neutrophils but also, in all probability, their maturation and proper function.

Macrophages intricately balance their more destructive attributes, their phagocytic capabilities utilized for debris removal and pro-inflammatory factor secretion to prevent infection (M1), with their anti-inflammatory capabilities for wound healing, angiogenesis, and remodeling (M2). This tenuous balance easily goes awry in many organs, including the eye, with fibrotic changes typically thought to be macrophage driven. In both Bcl-2^Flox/Flox^ and Bcl-2^MP^ mice, the initial levels of fibrosis (defined by collagen I staining) and CNV (Isolectin B4 staining) were similar at 2 weeks, consistent with similar levels of CD80^+^ myeloid cells (M1 marker) in these mouse CNV lesions. Decreased staining with the pan-MP marker F4/80 was noted in Bcl-2^MP^ mice lesions, with no accompanying early changes in M1 (CD80^+^) or M2 (CD206^+^) myeloid populations. However, microglial (Iba1^+^) cell staining, which also stained positively for F4/80, was significantly decreased in Bcl-2^MP^ mouse lesions following laser photocoagulation. Thus, early decreases in microglia and neutrophils corresponded with mitigation of later-stage fibrosis in Bcl-2^MP^ mouse lesions.

Later-stage fibrosis, which occurs after the initial inflammation has dwindled, is typically more rigid and demonstrates reduced elasticity, which in turn impacts organ function. In the eye, this rigid fibrotic scar leads to retraction and retinal detachment. Unfortunately, the processes involved are not well understood, leaving few ways to halt or reverse this damaging vision-threatening fibrosis. M2 macrophages are categorized as anti-inflammatory, and although they are involved in wound healing, they are typically considered pro-fibrotic. More studies are needed in this area to clarify their key functions in an organ-specific manner, particularly in the eye.

Studies in the kidney show the importance of M2 macrophages during the repair phase following injury, with their depletion hindering repair [42,43,44,45]. The data shown here correlate persistent maintenance of CD206^+^ myeloid cells (M2 marker), later during the repair process, with decreased later-stage fibrosis. Perhaps the lack of Bcl-2 in MP extends the repair phase, decreasing fibrosis, while the lack of Bim expression impairs this process. Thus, the ratio of Bcl-2 to Bim in MP aids in modulating later-stage fibrosis. However, the details of the molecular and cellular mechanisms involved require further investigation.

Subretinal fibrosis is a documented risk for poor vision outcomes associated with nAMD. Extended cell lifespan, through persistent Bcl-2 expression (tipping the balance away from Bim), has a well-documented negative impact on tissue homeostasis [46]. Bcl-2 extends the life span of macrophages and is a key contributor to fibrosis and scarring such as IPF and hypertrophic scarring [13,14,15]. In the case of IPF, new or established fibrosis from chronic inflammation due to decreased inflammatory cell loss is treatable with Bcl-2 inhibitors [14,47]. Many approaches to block Bcl-2 function and increase cell loss have been developed to successfully treat diseases such as cancer [36], including the FDA-approved Venetoclax (ABT199).

Given the critical role macrophages play in the fibrotic responses leading to subretinal scar formation, gaining a better understanding of whether decreasing the Bcl-2 to Bim ratio improves ocular fibrosis, when the clearance of cells is impaired, is critical to establish new and complementary therapeutics. Here, we showed significant decreases in the levels of CNV and fibrosis with ABT199 and Mcl-1/Bcl-2-IN-1 treatment following laser photocoagulation. ABT199 significantly decreases inflammation, demonstrated by enhanced clearance of F4/80^+^ macrophages/MP following laser photocoagulation in wild-type mice [35,36]. One caveat for the effective use of ABT199 is that Bim expression is required. This is nicely illustrated in Supplementary Figure S2, showing that in the absence of Bim in MP (Bim^MP^ mice), ABT199 does not impact clearance of F4/80^+^ cells following laser photocoagulation. These data further support the two-signal model delineated by Kurtulus et al., where downregulation of Bcl-2 is not sufficient to facilitate cell loss without Bim expression [20]. We also recently showed that polymorphisms or mutations that reduce Bim levels and/or activity could contribute to the noted lack of response to anti-VEGF treatment in patients with nAMD [48]. Most importantly, no vision issues have been reported as side-effects with the use of ABT199 in preclinical toxicity studies or clinical trials (https://clinicaltrials.gov/study/NCT02141282, accessed on 1 August 2026), giving promise for treating ocular disease without inducing further damage.

No pharmacological agents exist for the treatment of PVR. Inflammation and retinal fibrotic scar formation associated with PVR are purportedly intertwined. Given that our studies here demonstrated the need for cell clearance to repress inflammation and prevent scar formation, we examined whether modulating Bcl-2 and Bim expression in MP impacted fibrosis in the retina. Utilizing a mouse model of PVR, we showed that the lack of Bcl-2 expression in MP decreased PVR levels (defined by collagen I staining), while the lack of Bim expression increased fibrosis levels in this model. Furthermore, ABT199 also significantly decreased PVR levels. Since Bcl-2 and VEGF reciprocally induce each other’s expression, we assessed the impact of anti-VEGF treatment in this PVR model. We noted that anti-VEGF treatment reduced PVR levels in mice, consistent with previous studies showing that aflibercept prevented PVR in rabbits [49]. Thus, modulation of the ratio of Bcl-2 to Bim may be the missing link to squelch inflammation and decrease retinal scarring.

Several limitations of this study should be acknowledged. First, subretinal fibrosis was primarily assessed using collagen I staining. Although collagen I is a well-established marker of fibrotic remodeling, additional fibrosis-associated markers, including α-smooth muscle actin (α-SMA) and fibronectin, are also commonly used. Notably, studies demonstrating increased collagen I deposition in experimental subretinal fibrosis have similarly reported concurrent expression of α-SMA and fibronectin [50,51], supporting the validity of collagen I as an indicator of fibrosis in this context. Second, while our findings establish a role for Bcl-2 modulation in regulating subretinal fibrosis and associated inflammatory responses in established experimental models, they do not fully define the underlying molecular mechanisms. Further studies will be required to delineate the signaling pathways through which Bcl-2 deficiency or pharmacological inhibition influences inflammatory cell responses and fibrotic remodeling. A more detailed understanding of these mechanisms may provide additional insight into the therapeutic potential of targeting Bcl-2 in neovascular AMD-associated fibrosis.

In summary, although ocular diseases with a fibrotic component have a negative impact on vision, few, if any, treatments are available to stop their development or facilitate regression. The variety of tissues, initiating events, and number of fibrotic mediators make deciphering the pathways involved in ocular fibrosis and identifying treatment modalities challenging. Although much research is needed to address this, the ability to monitor outcomes by vision maintenance is beneficial. The studies presented here demonstrate the role of Bcl-2 in modulating ocular inflammation and fibrosis. Our studies demonstrated the efficacy of Bcl-2 inhibitors in curtailing ocular fibrosis associated with CNV and PVR. Thus, therapeutic strategies to enhance MP clearance may improve ocular disease outcomes in which fibrosis compromises vision.

## 4. Materials and Methods

### 4.1. Sex as a Biologival Variable

All experiments were performed using mixed-sex cohorts consisting of at least five male and five female mice per treatment group. Sex was included as a biological variable in all analyses, and no statistically significant sex-dependent differences were detected.

### 4.2. Mouse Studies

Conditional Bcl-2 mice (Bcl-2^Flox/Flox^; Stock #008882; Jackson Laboratory, Bar Harbor, ME, USA) were healthy and previously characterized [52,53,54]. Conditional Bim mice (obtained from Drs. Andreas Strasser and Phillipe Bouillet) were healthy and well characterized by us and others [17,19,55,56]. Conditional mice were crossed with lysozyme 2 (Lyz2)-Cre (Stock #004781; Jackson Laboratory [57]) mice to ultimately generate conditional mice that also expressed Lyz2-Cre (mononuclear-phagocyte (MP)-targeted mice called Bcl-2^MP^ or Bim^MP^) after multiple crossings [17]. However, Lyz2-Cre activity is not only restricted to MP. Genotyping was performed as previously described [17,53].

RD1 and RD8 mutations were not observed with screening. All mice were on a C57BL/6J background and were maintained at the University of Wisconsin animal facilities. C57BL/6J mice are at times referred to as wild-type (WT) mice in this manuscript. Generally, based on our long-term experience with the models used here, 10 mice per group were sufficient to provide the necessary power for statistical evaluations. All experiments were carried out with 12-week-old mice.

The experiments delineated here were performed in accordance with requirements of the Association for Research in Vision and Ophthalmology for utilizing animals in ophthalmic and vision research and were approved by the Institutional Animal Care and Use Committee of the University of Wisconsin School of Medicine and Public Health (IACUC assurance # D16-00239). All animal procedures were performed in accordance with the relevant institutional and national guidelines and regulations for the care and use of laboratory animals.

### 4.3. Mouse Laser Photocoagulation Model of Neovascularization and Subretinal Fibrosis

For laser photocoagulation studies, 12-week-old mice, male and/or female, were used (10 mice per group). Mouse pupils were dilated using a drop of 2.5% phenylephrine, followed by a drop of 0.5% tropicamide (Bausch and Lomb Inc., Bridgewater, NJ, USA). After pupil dilation, the mice were anesthetized with ketamine hydrochloride (90 mg/kg) and xylazine (10 mg/kg). We used an OcuLight GL diode laser fitted with a slit lamp delivery system (Iridex, Mountain View, CA, USA) to locate positions 3, 9, and 12 o’clock on the posterior pole of each eye. A handheld coverslip allowed us to view the retina and rupture of the Bruch’s membrane (75 µm spot size, 0.1 s duration, 120 mW). The levels of neovascularization and fibrosis, as well as recruitment of various inflammatory cells, were assessed at different times post-laser, as detailed in the figure legends, using captured fluorescence images and their quantitative assessments using ImageJ software (National Institute of Mental Health, Bethesda, MD, USA; http://rsb.info.nih.gov/ij/, accessed on 1 May 2026, version 1.54r), as detailed below.

### 4.4. Mouse Proliferative Vitreoretinopathy (PVR) Model

The dispase model of PVR in rabbits has been widely used to investigate the molecular and cellular mechanisms involved in the pathogenesis of PVR and the development and testing of potential treatments [58,59,60]. The development of such a model in mice, with potential for evaluating the significant impact of various genes and their regulatory pathways, has lagged behind [61,62,63]. We have developed and characterized a dispase PVR model in mice following rigorous optimization of conditions. For PVR studies, dispase (Worthington Biochemical Corporation, Lakewood, NJ, USA; LS02100) was prepared in PBS and administered (0.0015624 units in 1 µL/eye) by intravitreal injection. For each condition, 10 mice (3-month-old male and/or female) were used. One week later, the eyes were harvested, fixed in 4% PFA, and the levels of fibrosis were assessed by immunostaining of retinal flatmounts with anti-collagen I. Areas of the total retina and collagen I staining were manually outlined by tracing the borders of the retina and collagen I staining using the “Freehand Selections” tool in ImageJ, version 1.54r. The quantified area measurements were used to calculate the percentage of collagen I area relative to total retinal area.

### 4.5. Treatments of Mice Subjected to Laser Photocoagulation and PVR

ABT199 (Venetoclax) is a selective Bcl-2 inhibitor used to treat several hematopoietic malignancies. There are no recognized, common ocular toxicities associated with ABT-199. Eye toxicity has not emerged as a dose-limiting or characteristic adverse effect in preclinical toxicology studies or clinical trials (https://clinicaltrials.gov/study/NCT02141282, accessed on 1 August 2026). Thus, for ABT-199, the available preclinical toxicology data do not identify the eye as a target organ. Ophthalmic examinations and histopathologic evaluations performed during standard repeat-dose toxicology studies did not reveal consistent treatment-related ocular lesions. In contrast, the compound sold as MCL-1/BCL-2-IN-1 (CAS 2493256-46-3, also referred to as Nap-1 by some vendors) is a research tool compound, not a clinical or IND-stage drug. Publicly available information is limited primarily to screening data (IC_50_ ≈ 4.45 μM for MCL-1 and 3.18 μM for BCL-2), and there are no published GLP repeat-dose toxicology studies or regulatory toxicology reports describing ocular safety.

The dose and administration schedule used here were selected based on published studies demonstrating the efficacy and tolerability of the test compound in vivo, as well as our preliminary studies evaluating its effects in the experimental models used here [64,65,66,67,68]. The chosen dosing regimen was intended to achieve sustained target inhibition throughout the period of active inflammation and tissue remodeling while minimizing potential toxicity or off-target effects. The timing of drug administration was designed to coincide with the critical phases of disease development in the respective models. Initiation of treatment and the duration of dosing were selected to ensure adequate drug exposure during the period when inflammatory cell recruitment, activation, and fibrotic responses are known to occur. Thus, the treatment schedule was based on both the pharmacological properties of the compound and the temporal progression of pathology in the experimental models.

In some studies, male and/or female C57BL/6J mice received an intravitreal injection (1 µL) of either vehicle (straight DMSO) or ABT199 (100 µM stock prepared in straight DMSO; TargetMol, Wellesley Hills, MA, USA; #T2119) per eye on the day of laser photocoagulation and 7 days later. In studies utilizing Mcl-1/Bcl-2-IN-1 (MedChemExpress, Monmouth Junction, NJ, USA; #HY-129681, dual inhibitor), mice (male and/or female) received intraperitoneal (IP) injection (200 µL) of vehicle (10% DMSO, 30% PEG 400, 7.5% Tween 20, 7.5% Tween 80, and 45% sterile H_2_O) or 15 mg/kg Mcl-1/Bcl-2-IN-1 starting 2 days prior to laser photocoagulation (−2 day) and then subsequently on days +2, +6 and +10 following laser photocoagulation. To our knowledge, our study is the first evaluation of dual MCL-1/BCL-2-IN-1 inhibition in CNV-associated subretinal fibrosis. In Supplementary Figure S2, it is shown that ABT199 was prepared in the same vehicle (10% DMSO, 30% PEG 400, 7.5% Tween 20, 7.5% Tween 80, and 45% sterile H_2_O) for IP delivery, as detailed in the figure legend.

### 4.6. Sample Preparations and Biochemical Studies for Laser Photocoagulation Model

Animals were sacrificed by carbon dioxide (CO_2_) inhalation. The eyes were harvested, as noted, up to 5 weeks following laser photocoagulation, fixed in 4% paraformaldehyde (4 °C for 2 h), and washed three times in PBS. The eyes were then sectioned at the equator, and the anterior half, the vitreous, and the retina were removed. The choroid-RPE tissues were incubated with blocking buffer (20% normal goat serum and 5% fetal calf serum in 1x PBS) for 1 h; then, anti-collagen I (Southern Biotech #1310-01; 1:500 in 1x PBS with 20% normal goat serum and 20% fetal calf serum), anti-ICAM-2 (BD Pharmagen #553326; 1:500, BD Biosciences, San Diego, CA, USA), anti-F4/80 (eBioscience #14-4801-82: 1:500, Thermo Fisher Scientific, San Diego, CA, USA), anti-CD206 (Bio-Techne; #AF2535; 5 µg/mL, Bio-Techne Corporation, Minneapolis, MN, USA), anti-Gr1 (Invitrogen #53-5931-82; 1:500, Thermo Fisher Scientific, Waltham, MA, USA), CD11c (Invitrogen #14-0114-82; 1:500), anti-Iba1 (Invitrogen #PA5-27436, 1:500), anti-CCR2 ( Biossusa #bs-23026R; 1:100, Bioss Inc., Woburn, MA, USA), or anti-CD80 (eBioscience #11-0801-81; 1:500) was added overnight at 4 °C. The samples were washed and incubated with appropriate secondary antibodies (Jackson ImmunoResearch, West Grove, PA, USA; 1:500) for 2 h at room temperature. Slides were mounted, and images were captured in digital format using a Zeiss microscope (Axiophot equipped with AxioCam HRm camera; New York, NY, USA).

In some cases, 2 weeks following laser photocoagulation, the choroid-RPE was co-immunostained with Griffonia simplicifolia Lectin I isolectin B4-FITC (B4 lectin) (Vector Laboratories, Newark, CA, USA; #FL1201; diluted 1:100 in PBS containing 20% fetal calf serum, 20% normal goat serum) at 4 °C overnight. This staining was not suitable for samples collected 5 weeks after laser photocoagulation, and instead, ICAM-2 staining was used due to lack of vascular Isolectin B4 reactivity following extensive tissue remodeling. After washing with PBS, slides were mounted and viewed by fluorescence microscopy; images were captured in digital format using a Zeiss microscope (Axiophot equipped with AxioCam HRm camera). Image acquisition and quantitative analyses were performed using identical acquisition parameters within each experiment. Fluorescent images were imported into ImageJ, where the CNV lesion was selected by setting a threshold level of fluorescence and adjusting contrast and brightness to match the original image. The area of the CNV lesion was quantified using the “Analyze Particles” tool in ImageJ, version 1.54r. The quantified area measurement of CNV lesions in pixels was converted to μm^2^ by using the pixel-to-length ratio (1 μm/3.1 pixels) for the specific microscope and objective. All CNV lesions from eyes developing subretinal hemorrhage during laser application were excluded from data analysis.

### 4.7. Sample Preparations and Biochemical Studies for PVR Model

For PVR studies, one week after dispase treatment, animals were sacrificed by CO_2_ inhalation, and eyes were harvested and fixed in 4% PFA. Retinas were harvested and stained with desired antibodies, as previously described by us [69,70,71]. For anti-VEGF treatment, the mice received an intravitreal injection of mouse anti-VEGF (R&D Systems, Minneapolis, MN, USA; AF-493-SP), prepared in PBS and administered (25 ng in 1 μL), or vehicle 2 days prior to dispase injection. Eyes were harvested 1 week after dispase injection. For Bcl-2 inhibitor studies, dispase was drawn into a Hamilton syringe, followed by selected treatment such that mice received intravitreally either dispase (0.0015624 units in 1 μL): DMSO (1 μL) or dispase (0.0015624 units in 1 μL): ABT199 (100 μM; 1 μL). Since ABT199 (TargetMol, Wellesley Hills, MA, USA; #T2119; 10 mM in DMSO) has shown efficacy in the prevention and regression of lung fibrosis, 5 days later, the mice received a second dose of either vehicle (DMSO) or ABT199, as described above. Eyes were harvested 9 days later to allow time for the treatment to function. All eyes were fixed in 4% PFA. Following fixation, the retinas were dissected and stained with anti-collagen I, as described above. Slides were viewed by fluorescence microscopy, and images were captured in digital format using a Zeiss microscope (Axiophot equipped with AxioCam HRm camera). The stained areas were evaluated as described above, and the percentage of collagen I staining area relative to the total retinal area was calculated.

### 4.8. Statistical Analysis

Data are presented as mean ± standard deviation (mean ± SD). Each data point represents an individual lesion (CNV) and retina (PVR). The operator performing the quantitative image analysis was blinded to the sample identiy. In CNV studies, lesion areas exceeding two standard deviations from the mean were considered outliers. This was predefined and applied while blinded, and these outliers were excluded from the statistical evaluations.

Statistical analysis was performed using GraphPad Prism version 9 for Windows software (GraphPad Software, La Jolla, CA, USA), and figures were prepared using Adobe Illustrator (Adobe Illustrator 2025; Version 29.8.10). Comparisons between two groups were performed using an unpaired two-tailed Student’s *t*-test for normally distributed data, determined by the Shapiro–Wilk test. Sample sizes (number of mice) for each experiment are reported in the corresponding figure legends. However, due to the nature of CNV experiments, some mice and/or eyes are not included. A *p* value < 0.05 was considered statistically significant, and the exact *p* values are provided in the figure legends. All data points are shown in the graphs, and additional details regarding data collection and analysis are provided in the corresponding figure legends.

For mixed effect analysis, lesion size was log-transformed prior to analysis. Observations greater than two standard deviations above the within-group mean were considered outliers and were excluded, consistent with the prespecified exclusion criterion. Because multiple lesion-level observations were obtained from individual mice, group differences were evaluated using a linear mixed-effects model, with group and sex as fixed effects and a random intercept for mouse ID. The model was fit using restricted maximum likelihood, and fixed-effect inference used Satterthwaite-adjusted degrees of freedom. The original unpaired two-tailed Student’s *t*-test was retained as a sensitivity analysis and yielded the same overall conclusions.

## Data Availability

All the data generated and used in the current study are presented in the manuscript and Supplementary Materials.

## Supplementary Materials

The following supporting information can be downloaded at: https://www.mdpi.com/article/10.3390/ijms27167455/s1.
