# Supplementary material for: Bcl-2-Dependent Persistence of Mononuclear Phagocytes Promotes Ocular Fibrosis
Source: Int J Mol Sci. 2026 Aug 20;27(16):7455. doi: 10.3390/ijms27167455 (PMC13513839; doi:10.3390/ijms27167455)
Supplement: Supplementary file 1 [file ijms-27-07455-s001.zip › ijms-4459068-supplementary.pdf]

Supplementary Figures

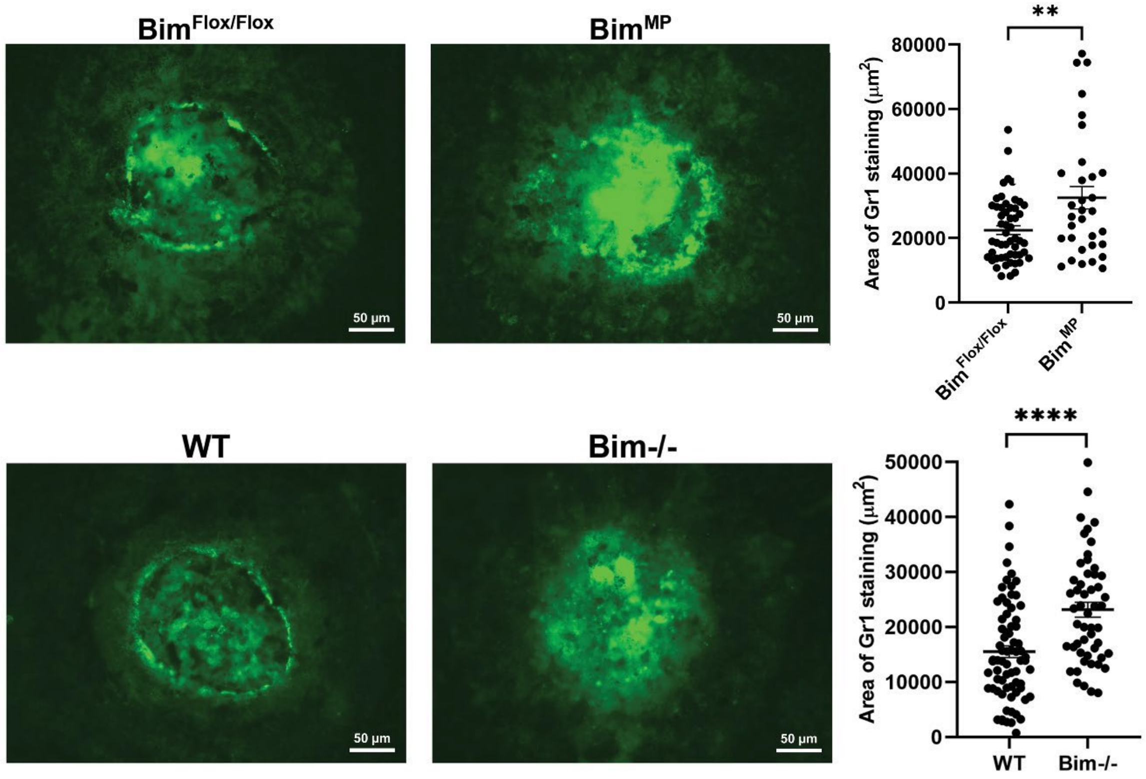

Supplementary Figure S1. Increased neutrophils ($Gr1^+$ cells) in the absence of Bim following laser photocoagulation. Three-month-old Bim transgenic (Bim^{MP} −MP-targeted and $Bim^{-/-}$−global KO) mice were subjected to laser photocoagulation. Three days later the choroid/RPE was harvested and stained with anti-Gr1. Images were captured using a Zeiss microscope and areas of staining were quantified by ImageJ. These experiments were performed with 10 mice for each condition. Scale bar = 50 μm. Quantitative data are presented as Mean ± SD. The statistical evaluations were performed as detailed in Methods. A *p*-value of less than 0.05 is considered significant. (** $p =$ 0.0045, **** p = 0.00005; respectively)

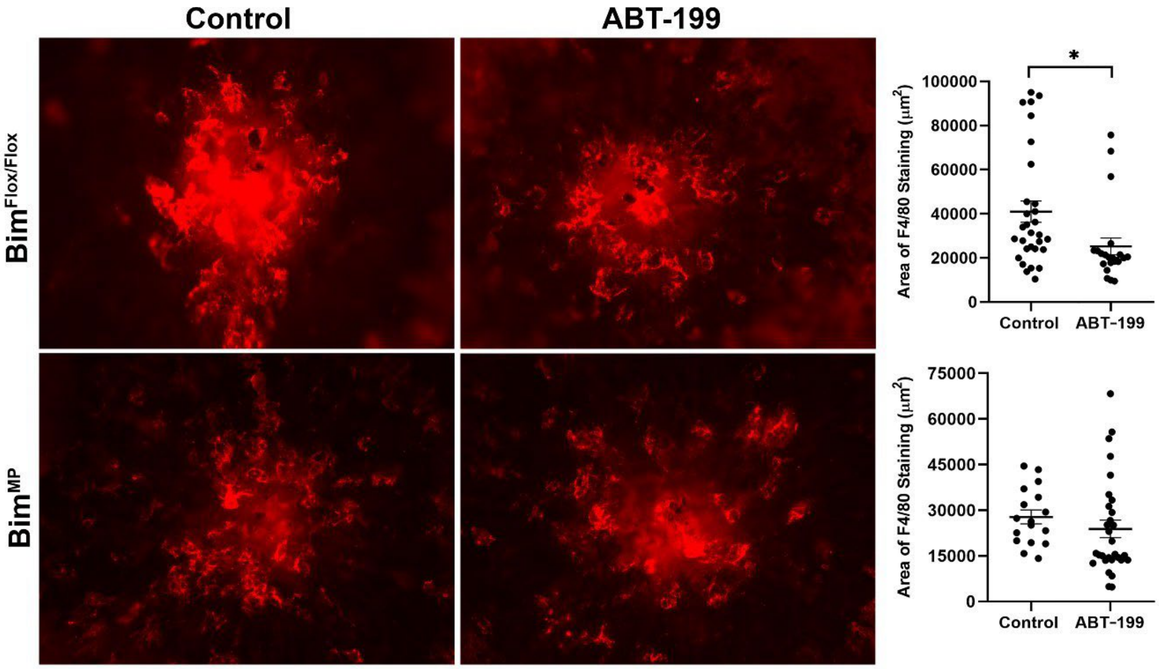

Supplementary Figure S2. ABT199 therapy decreases F4/80 staining in Bim$^{Flox/Flox}$ (wild type) mice. Male and female Bim$^{Flox/Flox}$ and Bim^{MP} mice were given intraperitoneal injections (0.2 ml of vehicle or 25 mg/kg ABT199) on Day -2 through Day 6. Vehicle was prepared in water (5% DMSO, 30% PEG400, 7.5% Tween 20, and 7.5% Tween 80). A stock solution of ABT199 (50 mg/ml in DMSO) was used to prepare the drug for daily IP injections using the vehicle solution. Laser photocoagulation occurred on Day 0. The choroid/RPE was harvested 6 days after laser and wholemount stained with anti-F4/80. Images were captured using a Zeiss microscope and areas of staining were quantified by ImageJ. These experiments were performed with 10 mice for each condition. Scale bar = 50 μm. Quantitative data are presented as Mean ± SD. The statistical evaluations were performed as detailed in Methods. A *p*-value of less than 0.05 is considered significant (* $p = 0.0189$; $p = 0.369$, respectively).
